# Janus kinase inhibition in the treatment and prevention of graft-versus-host disease

**DOI:** 10.3389/fimmu.2024.1304065

**Published:** 2024-02-06

**Authors:** Elisa De Togni, Oladipo Cole, Ramzi Abboud

**Affiliations:** ^1^ Department of Medicine, Washington University School of Medicine, St. Louis, MO, United States; ^2^ Division of Oncology, Washington University School of Medicine, St. Louis, MO, United States

**Keywords:** GvHD (graft-versus-host disease), JAK, Janus kinase (JAK), hematopoietic stem cell transplantation, acute GVHD, chronic GVHD, JAK inhibitor, ruxolitinib

## Abstract

Graft-versus-host disease (GVHD) is a significant cause of morbidity and mortality after allogeneic hematopoietic stem cell transplantation (HSCT). For many years, corticosteroids have been the mainstay treatment for GVHD, but cases of steroid-refractory GVHD and the severe adverse effects of high-dose corticosteroids have increased the need for preventative and therapeutic strategies for GVHD. Due to the nature of alloreactive T cells, GVHD is inherently linked to the graft-versus-leukemia (GVL) effect, the therapeutic driving force behind stem cell transplantation. A considerable clinical challenge is to preserve GVL while suppressing GVHD. The field of GVHD research has greatly expanded over the past decades, including advancements in T cell modulation and depletion, antibody therapies, chemotherapeutics, cellular therapies, and Janus kinase inhibition. In this review, we discuss current approaches and advances in the prophylaxis and treatment of GVHD with a focus on new emerging advancements in Janus kinase inhibitor therapy.

## 1 Introduction

Allogeneic hematopoietic stem cell transplantation (HSCT) is the only curative therapy for certain hematological conditions. Allo-HSCTs exert a therapeutic impact via both cytoreduction by the conditioning regimen and the graft versus leukemia (GVL) effect, in which donor immunocompetent alloreactive T cells from the graft attack leukemia cells in the recipient (1, 2). Unfortunately, the benefit of the GVL effect is counterbalanced by graft-versus-host disease (GVHD), a process similarly mediated by the donor alloreactive T cells attacking recipient tissue leading to major morbidity and mortality in the post-transplant setting. The Center for International Bone Marrow Transplant Registry (CIBMTR) assessed data from 2,254 patients undergoing matched sibling HSCTs for leukemia (CML, AML and ALL in first remission). After controlling for other factors affecting relapse, they showed a statistically significant decrease in relapse risk for patients who developed GVHD, especially chronic GVHD (1). In traditional transplantation approaches, the GVL effect and GVHD are closely intertwined, and identifying therapeutic targets to inhibit GVHD while preserving GVL remains a significant challenge.

GVHD occurs through a number of mechanisms, primarily by immunologically competent T cells from the donor graft reacting to foreign antigens expressed on recipient cells, triggering an immune response (3, 4). The activation of T cells to foreign antigens is driven by their interactions with extremely polymorphic proteins called human leukocyte antigens (HLA) expressed by major histocompatibility complex (MHC) and include class I HLA proteins (A, B, C) which are present on nearly all cells and class II HLA proteins (DR, DQ, DP) expressed on antigen presenting cells (APCs), such as dendritic cells, B cells, and monocytes (5). Not only are T cells activated via their receptors, but costimulatory pathways and cytokine production also play a large role in promoting T cell activation. The conditioning regimen of chemotherapy and radiation before transplantation damages recipient tissues, releasing cytokines such as IL-1, IL-6, and TNF-α causing systemic inflammation (6). Higher rates of acute and chronic GVHD have been found to occur with myeloablative regimens compared to reduced intensity or nonmyeloablative regimens (2, 7). Cytotoxic CD8 T cells from the donor graft are activated by recipient APCs, releasing further inflammatory cytokines such as IL-2, and IFN-γ. Activated cytotoxic donor T cells then target and cause damage to recipient tissues (8). Neutrophilic infiltration into tissues with release of reactive oxygen species and subsequent inflammation has also been found to be a key component of GVHD especially early on in its course (4).

Acute GVHD (aGVHD), traditionally described as occurring within the first 100 days post-transplant, affects multiple organ systems including the skin, GI tract, and liver. Clinical manifestations include a maculopapular or desquamating rash, hyperbilirubinemia, jaundice, nausea, vomiting, increased stool output, abdominal pain, and bloody stool. Acute GVHD occurs in around 30-50% of all patients undergoing allogeneic HSCT with 14% developing severe aGVHD (grades II-IV) (9, 10). A CIBMTR analysis including 4,224 AML patients and 1,517 MDS patients revealed that patients who developed aGVHD post-transplant had a higher risk of treatment related mortality (HR 2.51, 95% CI 2.18 – 2.89), and reduced overall survival (HR 1.71, 95% CI 1.55-1.89) (2). CIBMTR data from 2018-2020 showed that in adult matched related donor (MRD) HSCTs, GVHD accounted for 12% of all deaths within the first 100 days post-transplant as well as 12% of all deaths at or beyond 100 days post-transplant. In adult matched unrelated donor (MUD) HSCTs, GVHD accounted for 14% of all deaths within 100 days of transplant, and in adult haploidentical HSCTs (haplo-HSCTs), GVHD accounted for 7% of all deaths within 100 days of transplant (11).

Chronic GVHD (cGVHD) was historically defined as occurring post-day-100 after transplant. However, the NIH Consensus Conference in 2005 reestablished the definition of acute, chronic, and overlap GVHD to be based on distinct clinical syndromes rather than on chronicity alone (12). Chronic GVHD typically occurs due to long term immune dysfunction and inflammation and can manifest as skin rash, dry irritated eyes and mouth, liver injury, eosinophilia, GI and lung involvement, though can affect other areas of the body (12–14). The incidence of cGVHD at one year is around 20-40% and per CIBMTR data trends and has been steadily increasing in incidence since 1995 (28% from 1995 to 1999, 37% from 2004 to 2007) with around 20% of these patients having severe cGVHD and greater than 30% having moderate cGVHD. Chronic GVHD is a major contribution to morbidity and non-relapse mortality (NRM). In a CIBMTR analysis of patients undergoing allo-HSCT in 2004-2007, they found that in patients without cGVHD, NRM at 1-year, 3-years, and 5-years was 20%, 21%, and 22% respectively, while in patients with cGVHD these rates were 18%, 30%, and 37% respectively (13). In adult matched unrelated donor (MUD) HSCTs, GVHD accounted for 11% of all deaths at or beyond 100 days of transplant, and in adult haplo-HSCTs, GVHD accounted for 11% of all deaths at or beyond 100 days of transplant (11).

Acute and chronic GVHD remain a significant source of morbidity and mortality in patients and are a major challenge limiting the use of allo-HCT. Thus, discovering ways to diminish GVHD while preserving GVL is a critical objective in hematopoietic transplantation.

## 2 Pathophysiology of the JAK-STAT pathway in GVHD

Janus kinases are intracellular non-receptor tyrosine kinases involved in cytokine and growth factor mediated signal transduction through the Janus kinase/signal transducer and activator of transcription (JAK-STAT) pathway, playing a role in cellular proliferation and immune response. The JAK-STAT pathway was first discovered in the early 1990s when researchers were investigating interferon-induced transcription activation (15). JAKs wield a catalytic domain and an auto-regulatory kinase domain, thus its eponym of the dual-faced god in Roman mythology. When cytokine ligands bind to the extracellular receptor, the intracellular JAKs auto-phosphorylate each other, subsequently allowing them to recruit, phosphorylate and activate downstream STAT proteins which then dimerize. The STAT dimer then translocates into the nucleus and binds to promoter and enhancer DNA sequences leading to targeted gene transcription (16).

In mammals four JAKs (Jak1, Jak2, Jak3, and Tyk2) and seven STATs have been identified through which a wide variety of cytokines exert their signaling effects (17). In preclinical mouse studies, JAK1 knock-out mice are stunted at birth, lack response to cytokines including IL-2, IL-6, IL-10, IFN-α, and IFN-γ, and die perinatally (18). Knock-out of JAK2 in mice leads to embryonic fatality due to lack of erythropoiesis with tissues not responding to IL-2, IL-3, IFN-γ, erythropoietin, thrombopoietin, or granulocyte or macrophage colony-stimulating factors (19). While Jak1 and Jak2 are expressed in most tissues, Jak3 is expressed by lymphoid tissue. JAK3 knock-out mice are found to develop severe combined immunodeficiency (SCID) due to impairment in B cell development associated with IL-2, IL-4, IL-7, IL-9, and IL-15 receptor signaling (20–22). Mutations in JAK tyrosine kinases resulting in abnormal constitutive signaling or loss of function are associated with myeloproliferative neoplasms such as polycythemia vera, essential thrombocythemia, and myelofibrosis, and inflammatory diseases such as systemic lupus erythematosus, rheumatoid arthritis, and asthma as well as conditions of immune dysfunction (23, 24).

T cell activity is the driving force behind both the GVL effect and GVHD. Preclinical studies have shown that IFN-γ and IL-6 cytokine pathways and downstream JAK1 and JAK2 signaling are central to this process (25, 26). Common inflammatory cytokines upregulated in aGVHD are IL-1, IL-6 and IFN-γ. T cell activation by inflammatory cytokines is mediated mainly through JAK1/2 kinases. STAT1 and STAT3 are downstream of JAK1/2 and have been shown to be critically important to the development of aGVHD in mouse models (27, 28). JAK inhibitors thus have garnered a role in the preclinical and clinical settings for treatment and prevention of GVHD.

## 3 Current standards in GVHD prophylaxis

### 3.1 Calcineurin inhibitors and methotrexate or mycophenolate

Acute GVHD is mediated by alloreactive T cells. GVHD prophylaxis is aimed at targeting T cell suppression. One of the most widely utilized standard approaches for GVHD prophylaxis is calcineurin inhibitor therapy. Calcineurin inhibitors (CNIs), such as tacrolimus, sirolimus or cyclosporine, work by impairing IL-2 mediated activation and proliferation of T cells. They are used in combination with methotrexate (MTX) or mycophenolate mofetil (MMF). MTX is a dihydrofolate reductase inhibitor which reduces production of thymidylate and purines, suppressing T cell reactivity and proliferation. MMF is converted to its active metabolite mycophenolic acid, which inhibits inosine monophosphate dehydrogenase, suppressing purine synthesis in T cells. These work synergistically with CNIs to prevent GVHD. The disadvantage of MTX is that it is associated with delayed hematopoietic engraftment, higher risk of oral mucositis, GI, pulmonary, and renal toxicity. Thus, MMF has emerged as an alternative to avoid MTX related toxicity (29).

There remains significant variability between institutions on choice of GVHD prophylaxis regimens. Studies comparing different combinations of CNIs and MTX or MMF have shown that tacrolimus/MTX was associated with decreased rates of aGVHD compared to cyclosporine/MTX in MRDs and myeloablative MUDs (30, 31). Chhabra et al. showed that among MUDs, tacrolimus/MTX was associated with a lower risk of grades I-IV and grades III-IV aGVHD compared with cyclosporin/MMF but no significant difference in cGVHD, disease-free survival, or overall survival (OS). Tacrolimus/MTX had a 44% risk reduction in grades II-IV aGVHD and 48% risk reduction in grades III-IV aGVHD compared with cyclosporin/MMF (29).

In a phase III trial (NCT03959241), patients undergoing MRD, MUD, or 7/8 mismatched unrelated donor allo-HSCT were randomly assigned 1:1 to cyclophosphamide (Cy)-tacrolimus-MMF or to tacrolimus-MTX standard prophylaxis. Patients receiving Cy-tacrolimus-MMF had a higher GVHD-free relapse-free survival (GRFS) of 52.7% at one year compared with 34.9% in the standard tacrolimus-MTX group as well as less severe acute and chronic GVHD and higher rates of immunosuppression-free survival at one year (32).

### 3.2 Post-transplant cyclophosphamide and other agents

Cyclophosphamide (Cy) is a nitrogen mustard with potent anti-neoplastic effects achieved through alkylation. It is utilized in the management and treatment of various neoplasms, including multiple myeloma, sarcoma, and breast cancer. In 1954, Friedman and Seligman postulated its cytotoxic anti-tumor effect through *in vitro* studies, leading to its FDA approval as a cytotoxic anticancer agent in 1959 (33). Over time, Cy has gained multiple indications for cancer treatment, immune-mediated diseases, and recently for its pre- and post-conditioning therapies.

Post-transplant cyclophosphamide (PTCy) has emerged as an established strategy for reducing GVHD while preserving the GVL effect (34). PTCy is administered shortly after transplantation. By selectively depleting the alloreactive T cells responsible for GVHD, PTCy reduces the incidence and severity of acute and chronic GVHD. Additionally, PTCy’s immunomodulatory effects promote the development of regulatory T cells, which help promote immune tolerance and inhibit excessive immune responses (35–37). The use of PTCy has shown encouraging results, offering a potentially effective and well-tolerated approach to mitigating GVHD and improving the overall success of transplantation.

Haplo-HSCT broadens the donor pool for patients requiring treatment, encompassing both malignant and non-malignant conditions. Recent advancements in haplo-HSCT, building on PTCy/tacrolimus/MMF platforms, have shown promise. A study conducted by Li et al. examined 62 participants who underwent haplo-HSCT. Among them, 35 received a lower dose anti-thymocyte globulin (ATG) in conjunction with low-dose PTCy-based prophylaxis, while 27 were administered ATG-based regimens for GVHD prevention. At 180 days, the cumulative incidences of GVHD, grades II-IV, were 17.7% and 6.8%, respectively, in the low-dose ATG/PTCy group. In the low-dose ATG/PTCy group, the one-year OS and relapse-free survival (RFS) were 80.0% and 80.4%, respectively. These figures exceeded the rates seen in the ATG-based group, with an OS of 59.4% and an RFS of 62.0%. Multivariate analysis confirmed the low-dose ATG/PTCy-based regimen as a significant independent risk factor, lowering the risk of grades II-IV (HR = 0.357; P = 0.049) and grades III-IV aGVHD (HR = 0.190; P = 0.046) (38).

Sirolimus, a mechanistic target of rapamycin kinase (mTOR) inhibitor, is widely used in GVHF prophylaxis regimens. Benjayan and colleagues, tested the efficacy of MMF and sirolimus following PTCy as GVHD prophylaxis in haplo-HSCT. With 32 patients evaluated, the study aimed to demonstrate a decrease in the 100-day cumulative incidence of grades II-IV aGVHD to 20%, down from the established point of reference of 40% after haplo-HSCT using tacrolimus, PTCy, and MMF. After a median follow up of 16.1 months, the primary endpoint was achieved, with day 100 overall incidence of grades II-IV aGVHD standing at 18.8% (95% CI, 7.5% to 34.0%). The study noted no instances of graft failure. The one-year probability of moderate to severe cGVHD according to National Institutes of Health (NIH) criteria was 18.8% (95% CI, 7.4% to 34.0%), while NRM, relapse, disease-free survival, GVHD-free RFS, and OS were reported as 18.8%, 22.2%, 59.0%, 49.6%, and 71.7%, respectively. These findings indicate that GVHD prophylaxis employing sirolimus and MMF following PTCy effectively mitigates grades II-IV aGVHD post-haplo-HCT (39). Lazzari et al. retrospectively assessed the use of sirolimus in combination with PTCy +/- MMF in 242 AML patients myeloablative allo-HSCT in variable donor types and also found that sirolimus/PTCy +/- MMF was safe and effective in preventing acute and chronic GVHD in all donor types (40). In a meta-analysis including multiple RCT studies, the addition of sirolimus in GVHD prophylaxis regimens was an effective option for GVHD prophylaxis though found to be associated with higher thrombotic complications (41).

Although PTCy has become standard of care in GVHD prophylaxis, it has several disadvantages including secondary poor graft function and thrombocytopenia (42–44). It has also been associated with CMV reactivation as well as higher infection risk including BK cystitis, upper respiratory viruses such as adenovirus, and bacterial infections. In retrospective studies with PTCy in haplo-HSCT the incidence of CMV reactivation was as high as 42-69% and higher in studies using ATG at 74-85% (45–47). Several other studies have echoed similar positive outcomes, firmly establishing PTCy as a standard regimen for GVHD prophylaxis in haplo-SCT and increasing curative options for patients. Ongoing research aims to optimize PTCy dosing regimens, explore its combination with other agents such as JAK inhibitors, and further refine its application to maximize patient outcomes after transplant.

### 3.3 Summary of other advances in GVHD prophylaxis

Over the past several decades, research in GVHD prevention strategies has continued to expand, including advancements in T cell modulation and depletion, antibody and cellular therapies. Because GVHD is facilitated by donor T cell alloreactivity with host tissues, T cell depletion of the donor graft prior to transplant has been utilized as a method to limit the development of acute and chronic GVHD. In the 1960s, Bortin and Saltzstein showed that using fetal liver instead of bone marrow in murine models decreased the risk of GVHD due to lower levels of thymic immune cells (48). Earliest uses of T cell depletion in humans were in patients undergoing haplo-HSCT for severe combined immune deficiency (49). Since then, many different ex vivo and *in vivo* T cell depletion methods have been developed including T cell agglutination, anti-T cell specific antibodies, anti-thymocyte globulin (ATG), antibody-magnetic bead conjugates, photodepletion, selective T cell depletion, and immune modulation with T regulatory (Treg) cells (50).

There have also been advancements in the use of T cell costimulatory inhibition for GVHD prevention. In 2021, abatacept was approved by the FDA for prophylaxis of aGVHD coupled with a calcineurin inhibitor and methotrexate (51). Abatacept is a selective CD80/CD86 costimulation blocker on APCs, inhibiting interaction with CD28 on T cells. Sitagliptin also exerts inhibitory effects on T cell costimulation via a different mechanism, acting as an inhibitor of dipeptidyl peptidase 4, or CD26, a costimulatory transmembrane T cell receptor (52–54). Further advances are being made in targeting T cell trafficking as a method for GVHD prophylaxis. Vedolizumab, a humanized monoclonal antibody against α4β7 integrins on T cells has shown efficacy in a phase 3 RCT in preventing intestinal aGVHD by impairing T cell trafficking to the GI tract with a phase 3 RCT (55, 56). Maraviroc, an inhibitor of CCR5, a chemokine receptor involved in T cell trafficking, was also investigated but did not show superiority to standard of care with PTCy/Tacrolimus/MMF (57, 58).

Other approaches have included targeting cytokine signaling pathways, notably IL-6. Acute GVHD is associated with an inflammatory cytokine profile similar to that of cytokine release syndrome (CRS), a clinical syndrome described as fevers, vascular leak, hypotension, respiratory and renal insufficiency, neurotoxicity, and transient cardiomyopathy in the context of dysregulated immune response (59–61). It is defined by high levels of inflammatory cytokines, including IL-2, IL-6, IFN-γ, and elevations of C-reactive protein (CRP) and ferritin from over stimulation of the immune system (59, 60, 62–69). Given the central role of IL-6 in the pathophysiology of CRS, tocilizumab, a monoclonal antibody against IL-6 receptor, has become standard of care for management of CRS in multiple settings and is being explored for prevention of GVHD as well (60, 64, 70). In 2021 Kennedy et al. performed a phase 3 double blind RCT of the addition of tocilizumab vs placebo to cyclosporin/MTX GVHD prophylaxis in 10/10 HLA MSDs and MUDs and found that tocilizumab did not significantly decrease incidence of grades II-IV aGVHD at day +100 though decreasing trends were seen in MUDs. A non-statistically significant reduction in aGVHD with tocilizumab was mostly seen in moderate grade II disease rather than in severe grades III-IV where it is most clinically needed (71, 72).

## 4 JAK inhibition and GVHD preclinical studies

Ruxolitinib is a selective JAK1/JAK2 inhibitor that was originally approved for use in myelofibrosis and polycythemia vera. JAK1 and JAK2 are involved in regulation of signaling of cytokines involved in GVHD physiology including IL-2, IL-6, IL-12, IL-23, and IFNγ. Ruxolitinib acts by inhibiting cytokine-induced phosphorylation of STAT3 (73). In several preclinical studies, Choi et al. explored the role of JAK inhibitors in treatment and prevention of aGVHD. They demonstrated in fully-MHC mismatched murine allo-HSCT models that treatment with ruxolitinib led to reduced aGVHD through reduced expression of CXCR3 and less trafficking of donor T cells to GVHD target organs (74). They subsequently showed that knockout of IFNγR^-/-^ or the chemokine receptor CXCR3^-/-^ in donor T cells reduced trafficking of donor T cells into organs involved in GVHD (75).

Spoerl and colleagues conducted preclinical murine studies with ruxolitinib where they found that JAK1/JAK2 inhibition impaired proliferation of effector T cells, suppressed proinflammatory cytokines, decreased histopathological GVHD grade, and improved overall survival of mice with aGVHD. They demonstrated that ruxolitinib inhibited CD4+ T cells from differentiating into cells that produce IFNγ and IL-17A, cytokines involved in GVHD development. Furthermore, they found that ruxolitinib in patients undergoing allo-HSCT increased levels of FoxP3+ regulatory T cells which have been shown to play a role in immune tolerance (25).

In another preclinical study, Carniti et al. assessed the effect of JAK1/JAK2 inhibition with ruxolitinib in a murine model of fully MHC mismatched bone marrow transplant. They showed that ruxolitinib not only treated aGVHD in mice but also improved survival and lowered tumor burden suggesting preservation of the GVL effect. Ruxolitinib was associated with reduced CXCR3 expression, which was thought to contribute to decreased donor T cell infiltration into GVHD target organs (76).

In a murine model of sclerodermatous cGVHD, Ryu et al. found that mice treated with ruxolitinib had significantly reduced severity of skin findings both clinically and pathologically. There were also lower numbers of IFNγ- producing CD4+ T cells, macrophage/monocytes, effector cells as well as expansion of regulatory T cells. IFNγ and monocyte chemoattractant protein 1 (MCP-1) from CD11b+ macrophage/monocytes were suppressed. Ruxolitinib additionally decreased activation of STAT1 in immune effector cells. Overall, these findings suggested that JAK1/2 inhibition reduced GVHD via its suppression of IFNγ and MCP-1 production by T cells and macrophage/monocytes respectively (77).

In a fully MHC-mismatched transplant model, another JAK1/2 inhibitor called baricitinib, not only prevented GVHD but also reversed ongoing GVHD (74, 75). In preclinical models, baricitinib, compared with ruxolitinib, was associated with increased expansion of Tregs at earlier time points after allo-HSCT. This is mediated through baricitinib sparing JAK3-STAT5 signaling, increased EGFR signaling, decreased helper T cell differentiation, reduced allogeneic antigen presentation and costimulatory molecule expression on recipient APCs, and overall enhanced multi-lineage engraftment (78–80).

The selective JAK1 inhibitor, itacitinib, has less pan-JAK inhibition than ruxolitinib or baricitinib. In cell-based assays dependent on JAK2, itacitinib has IC50 values of approximately 1 μM or greater, suggesting that itacitinib is JAK2 sparing in cells (81). It inhibits the growth of the cytokine-dependent cell line INA-6. In *in vivo* models of JAK dependent malignancy, itacitinib impedes subcutaneous tumor growth of INA-6 cells expressing wild type JAKs when administered by continuous infusion, achieving plasma concentrations well below those necessary to inhibit JAK2. Oral itacitinib also modestly reduced splenomegaly in a murine model of JAK2 V617F–driven neoplasia relevant to MF though expectedly less than JAK2 inhibitors (82, 83). In pre-clinical murine models, itacitinib was found to prevent xenogeneic GVHD in humanized mice. Itacinitib-treated mice were found to have reduced numbers of human CD4+ and CD8+ T cells as well as increased numbers of human Tregs after transplant (84). Altogether, this body of work suggested that Altogether, this body of work suggested that JAK inhibitors may be effective in both prevention and treatment of GVHD to be investigated in subsequent clinical studies (**Table 1**).

**Table 1:** Janus kinase inhibitors FDA approved or under clinical investigation for hematologic oncologic disorders.

Agent	Target	Disease	Clinical Phase
FDA Approved			
Fedratinib	JAK2	MF	FDA Approved
Momelotinib	JAK1, JAK2	MF	FDA Approved
Pacritinib	JAK2, FLT3	MF	FDA Approved
Ruxolitinib	JAK1, JAK2	MPNs Steroid-refractory aGVHD Steroid-refractory cGVHD	FDA Approved
Orphan Drug			
Cerdulatinib	JAK, SYK	PTCL	Orphan Drug Approval Phase II (NCT04021082)
Lestaurtinib	JAK2	AML	Orphan Drug Approval Phase II (NCT00079482, NCT02428543)
Undergoing Investigation			
Baricitinib	JAK1, JAK2>JAK3, TYK2	cGVHD	Phase II (NCT02759731)
		aGVHD prophylaxis	Phase I (NCT04131738)
Cerdulatinib	JAK, SYK	CLL, SLL, NHL	Phase I/II (NCT01994382)
Gandotinib	JAK2	MPNs	Phase II (NCT01594723)
Itacitinib	JAK1	aGVHD	Phase I (NCT04070781, NCT03497273) Phase II (NCT03846479, NCT03721965) Phase III (NCT03109604)
		cGVHD	Phase II (NCT04200365) Phase III (NCT03584516)
		aGVHD prophylaxis	Phase I (NCT03320642, NCT03755414) Phase II (NCT04127721)
		CRS prophylaxis	Phase I/II (NCT04071366)
		MF	Phase II (NCT03144687)
		HL	Phase II (NCT03697408)
		ALL	Phase I (NCT03989466)
Momelotinib	JAK1, JAK2	MPNs	Phase III (NCT04173494)
Pacritinib	JAK2, FLT3	MPNs	Phase II (NCT03645824)
		Lymphoproliferative disorders	Phase I (NCT03601819)
		aGVHD prophylaxis	Phase I/II (NCT02891603)
Ruxolitinib	JAK1, JAK2	aGVHD prophylaxis	Phase I/II (NCT02806375, NCT02917096, NCT06008808, NCT05622318)
		AML, ALL, CML, HL, NHL, Hypereosinophilic disorders	Many trials ongoing

MF, myelofibrosis; MPN, myeloproliferative neoplasms; aGVHD, acute graft-versus-host disease; cGVHD, chronic graft-versus-host disease; PTCL, peripheral T cell lymphoma; AML, acute myeloid leukemia; CLL, chronic lymphocytic leukemia; SLL, small lymphocytic lymphoma; NHL, non-Hodgkins lymphoma; CRS, cytokine release syndrome; HL, Hodgkins lymphoma; ALL, acute lymphocytic leukemia.

## 5 JAK inhibitors for treatment of GVHD

### 5.1 Ruxolitinib

In 2014, Spoerl et al. performed early studies on the use of ruxolitinib in patients with steroid-refractory GVHD. After demonstrating that ruxolitinib abates GVHD and improves survival in murine models via increasing the number of T regs, they subsequently tested ruxolitinib in six patients with an initial dose of 5 mg twice daily followed by an increase to 10 mg twice daily if no side effects were seen after three days of the lower dose. Ruxolitinib decreased stool production in two of the patients who had intestinal GVHD, decreased bilirubin level in one patient with liver GVHD, and reduced skin GVHD in four patients from an involved area of 50% to less than 25% (25). In a retrospective study, Zeiser et al. looked at 95 patients (54 with grade III-IV aGVHD and 41 with moderate to severe cGVHD) receiving ruxolitinib for steroid-refractory GVHD across 19 different centers. The overall response rate (ORR) was 81.5% in steroid-refractory aGVHD with a complete response (CR) of 46% and the ORR was 85.4% in steroid-refractory cGVHD (85).

The REACH1 trial (NCT02953678) was a non-randomized, single cohort, open label phase 2 trial that studied the JAK1/JAK2 inhibitor ruxolitinib in patients with steroid-refractory acute and chronic GVHD. Patients 12 years and older undergoing allo-HSCT from any source with grade II-IV steroid-refractory aGVHD were included. Ruxolitinib was given at a dose of 5 mg twice daily with an optional dose increase to 10mg twice daily if there were no cytopenias. The primary endpoint was ORR, defined as complete, very good partial, or partial response. At day 28, ORR was 55% (95% CI 43-67%) regardless of GVHD grade (68% had grade III-IV GVHD), and median time to response was 7 days (ranging 6-49 days). The most common adverse events were anemia, hypokalemia, thrombocytopenia, neutropenia, and peripheral edema (86).

In 2019 ruxolitinib became the first FDA approved agent for treatment of steroid-refractory aGVHD after the REACH1 trial demonstrating safety and efficacy in steroid-refractory GVHD. This was quickly followed by subsequent phase 3 trials REACH2 (NCT02913261) and REACH3 (NCT03112603). In REACH2 (NCT02913261), a phase III multicenter RCT, 309 patients with steroid-refractory aGVHD were randomized to the ruxolitinib group (10 mg twice daily) or the control group (investigator’s choice of therapy from a list of commonly used options). The ORR at 28 days was 62% in the ruxolitinib group vs 39% in the control cohort (OR 2.64, 95% CI 1.65 to 4.22; P<0.001) and durable ORR at day 56 was 40% vs 22% respectively (OR 2.38; 95% CI, 1.43 to 3.94; P<0.001). The median OS was 11.1 months in the ruxolitinib group vs 6.5 months in the control group (HR 0.83, 95% CI 0.60 to 1.15). The most common adverse events included thrombocytopenia (33% vs 18% respectively), anemia (30% vs 28%), and CMV infection (26% vs 21%) (87).

REACH3 (NCT03112603) a randomized control phase 3 trial, subsequently looked at the use of ruxolitinib for treatment of cGVHD. A total of 329 patients with moderate or severe steroid-refractory or steroid-dependent cGVHD were randomized to the ruxolitinib (10 mg twice daily) or the control group (investigator’s choice). Week-24 ORR was 49.7% in the ruxolitinib group and 25.6% in the control group with ruxolitinib having greater median failure-free survival (>18.6 months versus 5.7 months respectively). Again the most common adverse events were thrombocytopenia and anemia. However, CMV infection and reactivation rates were similar between the groups (88).

### 5.2 Itacitinib

Itacitinib was studied in the steroid-naïve aGVHD setting in an open-label phase I trial. Twenty-nine patients undergoing first HCT from any donor source with grade IIb-IVd aGVHD were randomized 1:1 to receive itacitinib 200 mg daily or itacitinib 300 mg daily along with all patients receiving corticosteroids. The primary endpoint was safety and tolerability, and the secondary endpoint was ORR. The most common adverse events were diarrhea in 48% and anemia in 38%. The ORR at day 28 for patients receiving 200 mg daily was 78.6% and 66.7% in the 300 mg group. The ORR was 75.0% in patients with treatment-naïve aGVHD and 70.6% in patients with steroid-refractory aGVHD. Overall they demonstrated that itacitinib was safe, well tolerated, and showed promising efficacy in aGVHD patients (89).

In a phase II multicenter trial (NCT03846479), Etra et al. found that itacitinib monotherapy was safe and effective as first line treatment in low risk GVHD. They compared 70 patients with low risk GVHD treated with itacitinib 200 mg daily for 28 days (with those responding able to receive another 28-day cycle) with 140 matched control patients treated with the standard corticosteroid treatment. Within 7 days, the response rate for the itacitinib group was 81% versus 66% (p=0.02) for the steroid group. The day 28 response rate was overall similar between both groups (89% versus 86%) as well as occurrence of flares (11% versus 12%). Of note, infection rates within 90 days were significantly lower in the itacitinib group compared to the steroid group (27% versus 42%, p=0.04) with patients developing fewer viral and fungal infections. The main adverse event was cytopenias but was similar between both groups though the itacitinib group was found to have less severe leukopenia. There was no significant difference in NRM, relapse, OS, or cGVHD (90).

The selective JAK1 inhibitor, itacitinib was studied in the steroid-naïve aGVHD setting in GRAVITAS-301, a phase 3 randomized multicenter double-blind trial, for use in allo-HSCT patients with grades II-IV aGVHD who had received up to two days of steroids. The trial enrolled 439 patients who were randomized to receive either steroids in combination with itacitinib or steroids plus placebo. The ORR at day 28 was 74% (complete response of 53%) for itacitinib and 66% (complete response of 40%) for placebo (OR 1.45, 95% CI 0·96–2·20, p=0·078). Grade III or higher adverse events occurred at a rate of 86% in the itacitinib group and 82% in the placebo group which mostly included thrombocytopenia or significant platelet count reduction (36% in the itacitinib group versus 31% in the placebo group), neutropenia or significant neutrophil count reduction (23% versus 21% respectively), anemia (20% versus 12% respectively), and hyperglycemia (12% versus 13% respectively) (91). This study did not meet its prespecified efficacy endpoint and itacitinib has not been approved in the steroid-naïve aGVHD setting.

## 6 JAK inhibitors for GVHD prophylaxis

### 6.1 Ruxolitinib

Given the morbidity associated with both acute and chronic GVHD, improving existing GVHD prophylaxis platforms through the incorporation of JAK inhibitors is a natural next step forward. Morozova et al. used PTCy and ruxolitinib for GVHD prophylaxis after HSCT in patients with myelofibrosis. Their prospective pilot study has enrolled 20 patients to date undergoing MRD, MUD, or haplo-HSCTs. Patients received ruxolitinib at a dose of 45 mg/day on days -7 to -2 and then 15 mg/day on Days 5 to 100. The regimen was safe with acceptable toxicities. Engraftment was attained in 17 patients. Notably, the ruxolitinib dose was reduced to 10 mg/day in all but two patients secondary to poor graft function. However, they had encouraging rates of GVHD: 25% with grade II-IV aGVHD,15% with grade III-IV aGVHD, and 20% with moderate cGVHD, and no cases of severe cGVHD (92). Given the morbidity associated with both acute and chronic GVHD, improving existing GVHD prophylaxis platforms through the incorporation of JAK inhibitors is a natural step forward (**Table 2**).

**Table 2:** JAK inhibitors in GVHD prophylaxis—Essential characteristics of the included studies and patients.

First Author (year)	Journal	Sample Size	GVHD Prophylaxis	Condition-ing Regimen	Donor Type	Graft Source	Acute GVHD	Chronic GVHD	OS	Relapse
Ruxolitinib										
**Morozova (2020**) (92)	Acta Haematologica	20	Ruxolitinib + PTCy	RIC	MRD, MUD, haplo	PBSC	Grade II-IV 25%, grade III-IV 15%	moderate 20%, severe 0%	85% at 2 yr	5% at 2 yr
**Ali (2022) (** 93)	Blood Advances	18	Ruxolitinib + tacro/sirolimus	RIC	MRD, MUD	PBSC	Grade II-IV 17%, grade III-IV 11%	42% at 1 yr	77% at 1 yr	6% at 1 yr
**Defilipp (2023) (** 94)	Blood ASH Abstract	63	Ruxolitinib + tacro/MTX	RIC	MRD, MUD	PBSC	Grade II-IV 14%, grade III-IV 4.8% at 6 mo	27% at 1 yr	78% at 18 mo	27% at 18 mo
**Hobbs (2023) (** 95)	Blood ASH Abstract	43	Ruxolitinib + tacro/MTX	RIC	MRD, MUD	PBSC	Grade III-IV 2.4% at 6 mo	Moderate-severe 11% at 1 yr	86% at 1 yr	10% at 1 yr
Baricitinib										
**Schroeder (2022) (** 96)	Blood ASH Abstract	24	Baricitinib + tacro/MTX +/- ATG or PTCy/MMF/tacro	MAC or RIC	MRD, MUD	PBSC	Grade II-IV 33%, Grade III-IV 4% at day 100	46% at 1 yr	70% at 1 yr	21% at 1 yr
Pacritinib										
**Pidala (2021) (** 97)	Clinical Cancer Research	12	Pacritinib + sirolimus/tacro	MAC or RIC	MRD, MUD	PBSC	Grade II-IV 25% at day 100	Mild 25%	83%	8%
Itacitinib										
**Abboud** (2023) (98)	Blood ASH Abstract	42	Itacitinib + PTCy/tacro/MMF	MAC or RIC	Haplo	PBSC	Grade II 17.3%, Grade III-IV 0% at day 100	Moderate or severe 5% at 1 yr	80% at 1 yr	10% at 1 yr

yr, year; mo, month; GVHD, graft-versus-host disease; OS, overall survival; PTCy, post-transplant cyclophosphamide; tacro, tacrolimus; MTX, methotrexate; ATG, anti-thymocyte globulin; MMF, mycophenolate; RIC, reduced intensity conditioning; MAC, myeloablative conditioning; PBSC, peripheral blood stem cell.

In another pilot open-label study, ruxolitinib was used in the peri-transplantation period for patients with myelofibrosis undergoing allo-HSCT. It was given either at a dose of 5 mg or 10 mg twice daily with fludarabine/melphalan conditioning and sirolimus/tacrolimus GVHD prophylaxis. Cumulative incidence of grades II-IV aGVHD was 17% in the lower ruxolitinib dose group and 11% in the higher dose group. This showed that ruxolitinib was safe with higher doses associated with reduction in rates of GVHD (93).

Ruxolitinib has also been studied in combination with tacrolimus/MTX for prevention of aGVHD in platforms not including PTCy. In a phase II multicenter trial, ruxolitinib was used in patients with primary or secondary myelofibrosis pre-, during-, and post-HSCT. Forty-three patients undergoing reduced intensity conditioning peripheral blood HSCT were included with standard tacrolimus/MTX GVHD prophylaxis plus ruxolitinib. Sixty percent (n=27) patients were already receiving ruxolitinib prior to the study. Fifty-two patients had mutations in JAK2, CALR, MPL or ASXL1 at baseline, but by day 100 mutations were undetectable by next generation sequencing in all but a single patient. GVHD-free relapse-free survival (GRFS) was 74% at one year. The cumulative incidence of grades III-IV aGVHD was 2.4% at 6 months, and 1-year moderate to severe cGVHD was 11%. There was better OS in patients receiving ruxolitinib pre-transplant compared to those who did not but was not statistically significant. Adverse events included thrombocytopenia, neutropenia, and anemia similarly seen in other studies. The preliminary data from this trial suggested that ruxolitinib may be a clinically beneficial tool in acute and chronic GVHD prophylaxis especially with low rates of severe disease (95, 99).

Beyond the myelofibrosis setting, in a multicenter phase II trial, ruxolitinib was studied as maintenance for cGVHD along with tacrolimus/MTX GVHD prophylaxis. No patients received PTCy GVHD prophylaxis. This study enrolled older adults between the ages 60-80 with AML in first complete remission or MDS undergoing MRD or MUD reduced intensity conditioning allo-HSCT. Ruxolitinib could be started between days +30 and +100 if patients demonstrated donor engraftment, remission of disease, absence of progressive aGVHD, and recovery of blood counts; otherwise patients were ineligible for the study. Ruxolitinib was given at a dose of 10 mg twice daily in 28-day cycles for up to 24 cycles with a median start date of 45 days post-transplant and median number of cycles of 14 (ranging 1-24). The 1-year incidence of all cGVHD cases was 27%, while the 1-year incidence of cGVHD requiring systemic therapy was 8.4%. The 1-year GRFS was 70% (95% CI, 56-80). Although this study showed low rates of cGVHD with ruxolitinib therapy, it was limited by including only older adults with median age of 68 (ranging 61-79) (94).

### 6.2 Baricitinib

In a phase 1 study, 24 subjects undergoing MSD and MUD transplantation received baricitinib (JAK1/2 inhibitor) either 2 mg or 4 mg daily from days -3 to +100 followed by a drug taper as well as tacrolimus/MTX in the 2 mg cohort or PTCy in the 4mg cohort +/- thymoglobulin for MUD subjects. At time of initial analysis in 2022, the median follow-up was 320 days (ranging 63-368 days). The cumulative incidence of grades II-IV aGVHD was 33% (4 in the 2 mg cohort, 4 in the 4 mg cohort). The incidence at day +100 of grades III-IV aGVHD was 4% (1 in the 4 mg cohort) and the incidence at any point was 12.5% (3 total, 2 of which happened after baricitinib was tapered off). There were 11 cases of cGVHD (8 of which were moderate). There were no events of primary graft failure. With low incidence of aGVHD on baricitinib therapy, further investigation is in progress to more fully evaluate the efficacy of baricitinib in GVHD prophylaxis (96).

### 6.3 Pacritinib

Based on promising pre-clinical studies of combined JAK2 and mTOR inhibition reducing GVHD and increasing Treg activation in murine models, pacritinib, a JAK2/FLT3 inhibitor was combined with sirolimus and low dose tacrolimus for GVHD prophylaxis in patients undergoing MRD or MUD allo-HSCT in a phase I trial (NCT02891603). A total of 12 patients were included, with 6 patients each receiving either pacritinib 100 mg daily or 100 mg twice daily administered from day 0 to day +70 and then tapered off by day +100. Median follow-up was 18 months. In the lower dose group, two patients developed grade II-IV aGVHD (both with GI involvement) and one patient with grade I aGVHD. In the higher dose group, one patient developed grade II-IV aGVHD (steroid-refractory skin and GI involvement after tacrolimus was stopped early for non-thrombotic microangiopathy related kidney injury) and three patients with grade I aGVHD. There were two patients, one in each dose group, who developed mild cGVHD neither of which required systemic immunosuppressive therapy. There was only one case of CMV reactivation in the lower dose group and no events of CMV reactivation or infection in the higher dose group (97).

### 6.4 Itacitinib

As a JAK1 selective inhibitor, itacitinib is also under active investigation for prophylaxis of GVHD and may be effective at preventing both GVHD and CRS without increasing incidence of engraftment failure compared to other less selective JAK inhibitors. An ongoing study assessing JAK1 selective inhibition with itacitinib for prophylaxis of GVHD and CRS in haplo-HSCT utilized itacitinib at doses of 200 mg/day on days -3 through +100 in combination with tacrolimus, MMF, and PTCy. Forty-two patients were enrolled and underwent haplo-HSCT with zero cases of grades III-IV aGVHD, incidence of grade II aGVHD of 17.3% at day +100, 20.5% at day +180, and 20.5% at one year. The cumulative incidence of moderate to severe cGVHD was 5% (95% CI, 1-17). Day +180 GRFS was 85% (95% CI, 69-93), and 1-year GRFS was 79% (95% CI, 62-89). There were no cases of severe CRS (typically occurring at a rate of 17%). No systemic steroid therapy or anti-IL6R therapy was used during the study, and there were no cases of engraftment failure. Additional analysis of peripheral blood mononuclear cells on days +28, +60, and +100 showed that relative to control haplo-HSCT subjects, subjects receiving itacitinib had lower levels of CD4 and CD8 memory and naïve T cells as well as elevated levels of monocytes and myeloid and plasmacytoid dendritic cells. They found that in patients treated with itacitinib, T cell expression of TIGIT was higher while that of LAG3 and PD-1 was lower. Monocyte expression of HLA-DR, CD80, and CD86 was also higher. Overall, this study demonstrated that itacitinib was safe and effective in lowering rates of acute and chronic GVHD without increasing risk of relapse or transplant related mortality (98, 100).

Clinical trials are currently ongoing to further investigate the effect of JAK inhibition with itacitinib in combination with PTCy for GVHD prophylaxis and prevention of CRS after haplo-HSCT as well as a phase II study adding itacitinib to tacrolimus/sirolimus GVHD prophylaxis after fludarabine/melphalan conditioning regimen which will continue to provide interesting insights into new therapeutic combinations for GVHD prophylaxis (98, 101).

## 7 Discussion

Allogeneic hematopoietic stem cell transplantation remains a cornerstone in treatment for patients with hematologic malignancies, and in many cases represents the best or only chance for long-term survival. Continued progress, including improvements in supportive care and the development of reduced intensity conditioning regimens have made allo-HCT available to older patients and those with medical comorbidities. Furthermore, increased use of haploidentical and mismatched unrelated donors have increased the donor pool to the point that almost all patients will have an available donor. Unfortunately, acute and chronic GVHD remain important complications of the procedure, limiting the benefit in some patients, even in the context of durable remissions. Novel approaches are needed, both for prevention and treatment of GVHD.

With preclinical studies in the last decade expanding our understanding of the role of cell signaling and T cell biology in the pathophysiology of GVHD, new therapeutic targets have been recognized and developed, notably JAK inhibitors. JAK inhibitors were first developed for treatment of GVHD. Ruxolitinib gained FDA approval for treatment of steroid-refractory GVHD and chronic GVHD and has since gained widespread use. It has a favorable side effect profile, with cytopenias being the main limiting toxicity. JAK inhibitors are now being explored in the setting of GVHD prevention with promising early clinical experience. However, many questions remain. With multiple recent FDA approvals for both acute and chronic GVHD, the optimal sequence of these therapies is unknown, and certain GVHD manifestations may favor one agent over others. It is also unclear how best to combine JAK inhibitors with existing GVHD therapies. In the real-world setting, ruxolitinib is often combined with other immunosuppressants, such as corticosteroids, calcineurin inhibitors, mycophenolate, belumosudil and extracorporeal photopheresis, although there is a paucity of prospective clinic trials to guide the use of these combinations (102, 103). Continued development of novel therapeutics for treatment of GVHD remains essential. However, the field must also focus on proper sequencing and combinations of existing agents.

Our patients continue to benefit from advances in the treatment of GVHD, but prevention of severe aGVHD and extensive cGVHD remain paramount. GVHD prophylaxis regimens have long incorporated multiple agents which suppress T cell activation in the peri-transplant period. JAK inhibitors represent an attractive add-on to these established regimens. Pre-clinical and early clinical studies have shown that JAK inhibitors can be safely added to GVHD prophylaxis platforms peri-transplant–itacitinib in the peripheral blood haploidentical PTCy setting, baricitinib in the peripheral blood matched donor setting with and without PTCy, and ruxolitinib in the myelofibrosis matched donor setting. Early clinical results are extremely promising, but much remains unknown.

How much does graft source, bone marrow versus peripheral blood, matter? Probably a significant amount. Should PTCy, abatacept, or both be prioritized in developing JAK inhibitor containing platforms? There are reasons to use both. Are calcineurin-free platforms possible (we believe they are), or should we focus on reducing dose and duration of CNIs? Going forward, it will be vital to avoid the mistakes of 1) assuming that JAK inhibitors are all the same, as there are differentiating properties between itacitinib, baricitinib, ruxolitinib, and others, and 2) that a JAK inhibitor can be used at the same dose and schedule in all donor types and GVHD prophylaxis platforms. It is unlikely that a single JAK inhibitor and dosing schedule will be optimal in the extremely varied and divergent allo-HSCT platforms in use. Itacitinib may cause less bone marrow suppression and allow for increased dose intensity compared with broader JAK inhibitors. Ruxolitinib may be more efficacious, given its proven track record in GVHD treatment. Baricitinib may have the best kinase inhibition profile, allowing for robust regulatory T cell expansion.

These questions highlight the importance of preclinical work in this field. Differentiating distinct effects of various JAK inhibitors on immune reconstitution after allo-HCT will help inform the decision on which JAK inhibitors are best suited to GVHD prevention, as opposed to treatment. It will also lead us towards rational partners–PTCy, abatacept, MMF, etc.—which may have synergistic effects on GVHD prevention without overlapping toxicity and help prioritize platforms to take forward into clinical trials.

JAK inhibitors are an attractive class of medications with a large body of clinical experience in malignant and nonmalignant diseases. They have become fundamental in the treatment of GVHD and soon to become so in the prevention of GVHD. Many questions remain, and both preclinical work and rationally designed clinical studies will be needed to maximize benefit of our patients.

## Author contributions

ED: Writing – original draft, Writing – review & editing. OC: Writing – original draft, Writing – review & editing. RA: Conceptualization, Supervision, Writing – review & editing.
